# Determination of Forming Limits in Sheet Metal Forming Using Deep Learning

**DOI:** 10.3390/ma12071051

**Published:** 2019-03-30

**Authors:** Christian Jaremenko, Nishant Ravikumar, Emanuela Affronti, Marion Merklein, Andreas Maier

**Affiliations:** 1 Pattern Recognition Lab, Friedrich-Alexander-Universität Erlangen-Nürnberg Martensstr. 3, 91058 Erlangen, Germany; nishant.ravikumar@fau.de (N.R.); Andreas.Maier@fau.de (A.M.); 2 Institute of Manufacturing Technology, Friedrich-Alexander-Universität Erlangen-Nürnberg Egerlandstr. 13, 91058 Erlangen, Germany; Emanuela.Affronti@fau.de (E.A.); Marion.Merklein@fau.de (M.M.)

**Keywords:** pattern recognition, machine learning, deep learning, forming limit curve, sheet metal forming

## Abstract

The forming limit curve (FLC) is used to model the onset of sheet metal instability during forming processes e.g., in the area of finite element analysis, and is usually determined by evaluation of strain distributions, derived with optical measurement systems during Nakajima tests. Current methods comprise of the standardized DIN EN ISO 12004-2 or time-dependent approaches that heuristically limit the evaluation area to a fraction of the available information and show weaknesses in the context of brittle materials without a pronounced necking phase. To address these limitations, supervised and unsupervised pattern recognition methods were introduced recently. However, these approaches are still dependent on prior knowledge, time, and localization information. This study overcomes these limitations by adopting a Siamese convolutional neural network (CNN), as a feature extractor. Suitable features are automatically learned using the extreme cases of the homogeneous and inhomogeneous forming phase in a supervised setup. Using robust Student’s t mixture models, the learned features are clustered into three distributions in an unsupervised manner that cover the complete forming process. Due to the location and time independency of the method, the knowledge learned from formed specimen up until fracture can be transferred on to other forming processes that were prematurely stopped and assessed using metallographic examinations, enabling probabilistic cluster membership assignments for each frame of the forming sequence. The generalization of the method to unseen materials is evaluated in multiple experiments, and additionally tested on an aluminum alloy AA5182, which is characterized by Portevin-LE Chatlier effects.

## 1. Introduction

The forming range of sheet metal is evaluated using the forming limit curve (FLC), whose limits are defined by the major and minor strain pairs at the onset of localized necking. In Europe, the procedure used for the generation of the FLC is summarized in DIN EN ISO 12004-2 [1]. The FLC is generated by means of Nakajima [2] and Marciniak [3] setups. The sheet metal is mounted into a clamping unit and then deformed to fracture by using a flat shaped or a hemispherical punch. The evaluation method proposed in the ISO standard is based on the Bragard study of 1972 [4], which evaluates the strain distribution of the material prior to failure and is referred to as the “cross-sectional method”. To determine the limiting strains, sections perpendicular to crack initiation are defined and the strain propagation is approximated with a second order polynomial. However, such an approach only allows evaluation of the last stage of the strain distribution without considering strain progression. Presently, the forming behavior is evaluated using digital image correlation techniques (DIC) and employed to determine the FLC [5]. For this purpose, a stereo camera system measures strains on the material surface during Nakajima or Marciniak tests and thus enables the evaluation of strain progression. Despite technological progress, the standard is still based on the “cross-section method”. An added complexity in determining the forming limits is related to the intrinsic properties of the materials, for example, modern lightweight materials such as high-strength steels and aluminum alloys tend to develop multiple local strain maxima or spontaneous crack initiation without a necking phase. Consequently, second order functions are unsuitable to approximate the limit strains. To overcome the disadvantages of the “cross-section method”, several techniques have been developed which take into account the forming history during the Nakajima tests. These time-dependent methods include the “line-fit” approach proposed by Volk et al. [6] as well as the correlation coefficient method [7]. Both approaches are based on observing the reduction in thickness within a predefined instability zone and determining the initiation of necking as a response to a sudden decrease in the thickness of the specimen. One disadvantage of these time-dependent methods is their dependence on a predefined instability zone and thus limited area of evaluation. In 2015, an approach based on machine learning was considered to provide new insights to forming development [8]. Conventional pattern recognition [9] involves the automatic processing and evaluation of data, whereby a physical signal, e.g., images or speech, is first converted into suitable, more compact characteristic features that are commonly defined by experts of the respective field. To enable an automated separation into subspaces/classes, the data is first assigned to different classes by experts. Using a classification algorithm and a representative subset of the data, decision boundaries are learned based on the feature representation/label pairs, such that the hypothesis of the learned class boundaries can be verified with the remaining disjoint data. In [8], such a pattern recognition technique was used to predict the crack class for a DX54D steel with a prediction accuracy of 90% before the actual crack formation was observed.

Metallographic investigations have shown that both conventional deep drawing steels [10] and dual phase steels [11] have superficial patterns associated with local necking, which enables the application of automated pattern recognition methods using the strain distributions for the determination of forming limits [12]. In [13] (henceforth referred to as Part 1) this idea was extended by the assessment of several evaluation areas and by comparing the obtained classification results with expert annotations for several failure classes (diffuse, local necking, and crack). This study demonstrated that a consistent determination of local necking is possible with the use of expert knowledge. Additionally, it was shown that the accuracy and efficacy of the results are affected by subjective expert annotations as well as external factors such as sampling frequency, which have a particularly negative effect on the classification results of the diffuse necking class.

To address these problems and to remove the dependence on expert annotations, an unsupervised classification approach was developed (henceforth referred to as Part 2) in [14]. Several rectangular areas close to the strain maximum were evaluated and Histogram of Oriented Gradients (HoG) were used as characteristic features [15], to classify the local necking using One-Class Support Vector Machines (SVM) [16]. It was shown that the results are within the range of the “line-fit” method, while incorporating image information and ameliorating the need for defining a specific evaluation area. Additionally, advantages of this approach include independence from expert annotations, transferability to new materials, and the introduction of a probabilistic FLC.

On the other hand, the conditional location dependence, i.e., the choice of the evaluation areas is still dependent on the strain maxima, which can be a disadvantage. Furthermore, the criteria used to determine the local necking are constrained by expert a priori knowledge, namely, the evaluation of HoG features, which are not necessarily applicable to other materials. Additionally, the assumption and partitioning of the forming process into a homogeneous and an inhomogeneous forming area can influence the outcome of the probabilistic FLC, if using another split criterion then approximately 50% of the sequence length.

In recent years, convolutional neural networks (CNN) have found widespread use in the field of pattern recognition, and can process a large amount of data in finite time due to the computing capacity that is now available, e.g., due to powerful graphics cards. The main advantage of these approaches is the automated, data-driven learning of representative features that are adapted to the problem, such that the design of handcrafted features is no longer necessary for the evaluation and assessment of classification problems. Different CNN approaches have outperformed conventional pattern recognition methods in various challenges (e.g., ILSVRC [17]) and are being successfully applied in the medical field, e.g., computer aided diagnosis [18], non-rigid registration [19], reconstruction [20,21], landmark detection [22], and in mechanical engineering for example in the field of event detection [23] or defect detection for photo-voltaic module cells [24].

In contrast to Part 1 and 2, this study uses a CNN for the automatic extraction of characteristic features, for the task of FLC estimation. Here, the extreme cases of the forming process (beginning of the homogeneous and end of the inhomogeneous forming phase) are used to train a Siamese CNN [25] to optimally separate the respective forming phases from each other. This allows the characteristic features to be adapted to the respective material properties, to enable a more precise description of the failure states and by extension, the estimation of a more accurate FLC. As a further advantage, time independency is introduced by clustering the feature representations of images using Students t Mixture Models [26], so that video sequences of incomplete forming processes may also be investigated, for e.g., prior to the occurrence of fracture. This not only allows a determination of forming limits independent of strain paths, which use the mean value of a heuristically defined region, but also the generation of the probabilistic FLC as introduced in Part 2, and theoretically a real-time monitoring of forming processes. To enable comparisons with previous studies, the data set of Part 1 is used, and the temporal derivative according to Part 2 is evaluated, which emphasizes the strain development over a sequence of successive images, as suggested by Vacher et al. [27]. In addition, the generalizability and transferability of the methodology to other materials that remain unseen during learning will be investigated. The data set consists of a deep drawing steel DX54D of two different thicknesses, a dual phase steel DP800, an aluminum alloy AA6014 (in previous studies referred to as AC170) and additionally another aluminum alloy AA5182 with Portevin-LE Chatlier effects (PLC) [28]. The results of the CNN approach are compared with the results of the method proposed in Part 2 and with the results of the time-dependent FLC of the “line-fit” method. Additionally, the transferability of the approach to unseen data is demonstrated by assessing forming tests which were stopped before fracture occurrence and for which metallographic examination results are available.

## 2. Experimental Procedure and Materials

**Figure 1:**
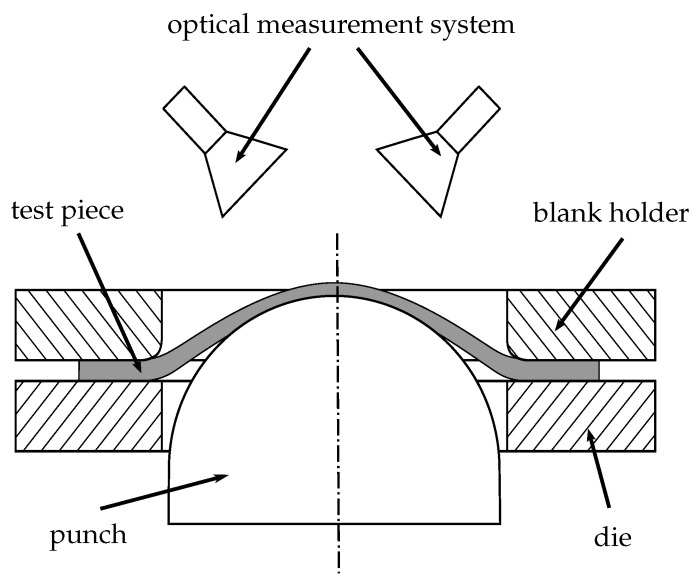
Schematic of the Nakajima experimental setup.

The FLC is usually determined experimentally with a Nakajima test setup, coupled with a measurement system that consists of a stereo camera, a clamping unit with an inner die diameter of 110 mm and a hemispherical punch with a diameter of 100 mm (cf. Figure 1). To calculate the strain distributions, a forming process is recorded by means of an optical measuring system (ARAMIS gom GmbH) and further processed using block matching (DIC) approaches. These methods require the specimens to be prepared with a white primer and a speckle pattern of black graphite to enable image correlation. A lubrication system according to DIN EN ISO-12004-2 is used to minimize the friction between the punch and the specimen to initiate a rupture at the top of the specimen. With a variation of the sample geometry to be investigated, starting from an uncut sample geometry up to conical blanks with parallel connections, induce different loading conditions and strain paths. The remaining conjunction width determines the name of the geometry, e.g., 50 mm corresponds to S050. For each sample geometry, three forming experiments are carried out and recorded. The test parameters are varied across the investigated materials (cf. Table 1), e.g., punching speed (1 to 2 mm/s) and sampling rate (15, 20, 40 Hz), in order to evaluate different boundary conditions to provide varying combinations of strain paths, and to test the generalization of the method to multiple loading conditions as uniaxial, plane-strain, or biaxial.

In this study, the three known materials from Part 1 and 2 are reused, while a challenging aluminum alloy is also evaluated: (1) DX54D, a deep drawing steel, with ductile necking behavior and observable localization on the surface; (2) DP800, a dual phase steel, high-strength material with a matrix of ferrite and martensite precipitations and hence multiple observable local maxima during Nakajima tests; (3) AA6014, a lightweight aluminum alloy of the 6xxx series with multiple maxima during Nakajima tests under plain strain conditions and (4) AA5182, an aluminum alloy of the 5xxx series with several local maxima in the form of shear bands (PLC effect). The principal material properties are summarized in Table 1. For a comprehensive description of the forming behavior of the evaluated materials please refer to Part 1.

## 3. Method

The proposed classification approach mainly follows the typical processing pipeline used in pattern recognition solutions, even though it uses a CNN. The conventional pattern recognition pipeline consists of four steps: data acquisition, preprocessing, feature extraction, and classification. The main difference, introduced with the advent of deep learning techniques, is the data-driven feature extraction and combination with the classification step. Instead of predefining the extracted features and employing these to train and test a classifier as in conventional pattern recognition systems, the feature extraction and classification steps are combined and formulated as optimization problem. Solving this optimization problem enables estimation of a problem-specific optimal solution, e.g., by minimizing a suitable problem-specific loss function. Comparable to the conventional pattern recognition approaches, the data set is subdivided into multiple disjoint data sets. During training of the network, the training data set is used to minimize the loss function, by adjusting the weights of the layers of the CNN using a gradient descent optimizer. After the network has seen all the training data, the separation hypothesis is evaluated using the validation data set to assess if the hypothesis generalizes to unseen data or if it over-fits to the training data. This procedure is repeated until convergence or until the error in the validation data set increases, and subsequently, the performance of the network is assessed using the remaining unseen test data set. There exist supervised and unsupervised deep learning-based techniques, similar to conventional pattern recognition approaches that either require expert annotations or solve the optimization problem without the use of data (class-) labels. The framework proposed in this study employs both types of learning techniques. In the supervised part, the extreme cases of the homogeneous and inhomogeneous forming phase, are used as labels for the data set. The number of images used per material for training are found in Table 2, where # images depicts the total number of images per sequence for the individual material and geometry, # homog. the number of images of the homogeneous forming phase and # neck. the number of images of the inhomogeneous forming phase. Only these extreme cases e.g., beginning 20 from the clear homogeneous and last 3 images from the inhomogeneous phase, close to fracture, are used to train two identical CNN’s that share the same weights and hence assess the similarity of the images. Such a setup was designed to account for the difficulty in assigning labels in a reliable manner to images in a sequence. Consequently, we adopt a Siamese CNN to extract features from images which are guaranteed to belong to either the homogeneous or inhomogeneous class. This setup enables pairwise comparisons between the two types of images using a similarity metric in a low-dimensional feature space. As a result, of the similarity metric, the two instances either belong to the same or different class. This supervised classification setup is used to separate the two classes in an optimal manner, by simultaneously learning compact representations of both classes.

In the unsupervised part, the unused data between the beginning of the homogeneous and the end of the inhomogeneous forming phase, as well as the extreme cases are assessed using the trained network to create low-dimensional representations. Using an unsupervised clustering approach, the low-dimensional instances of each forming sequence are then assigned to one of the three clusters: homogeneous, transition, necking. For clarification, the data sets are disjoint, meaning that e.g., the extreme cases of all but one material are used for training, and validation as well as to learn an optimal separation between the two classes and a low-dimensional representation of the data. The actual evaluation and clustering is performed using the held-out material.

### 3.1. Preprocessing

The study uses the data set of Part 1 & 2 and was acquired using the ARAMIS optical measuring system (v6.3.0-7), and consists of three-channel video sequences comprising of major and minor strain as well as thinning. The difference between two successive images were used as input to remove the correlation with the punch displacement and to emphasize the incremental changes. Since the necking and in general the material instabilities are localized changes in the strain distribution, the incremental changes allow a better evaluation of this phenomenon. The sequences were adjusted by removing the frames that contain the fracture information, identified at the end of the forming process with an increasing amount of defect pixels due to specimen cancellation.

**Figure 2:**
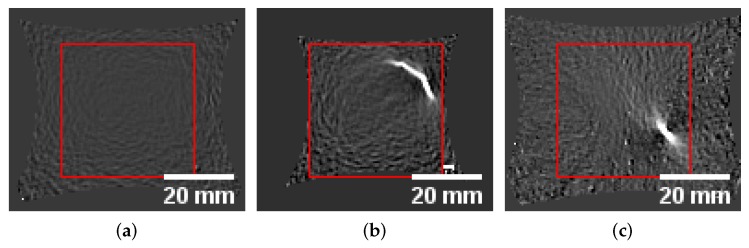
Varying images sizes depending on forming progression. Center cropped evaluation areas of DX54D and DP800 showing the extreme cases of the forming process: (**a**) DX54D-S245 homogeneous. (**b**) DX54D-S245 localization. (**c**) DP800-S245 localization.

Single defect pixels or missing values that occurred occasionally were interpolated linearly using the temporal information, e.g., the strain progression. Other defect pixels, such as static defect pixels that contain no information during the whole forming process were replaced by the average value of a 3×3 neighborhood. To reduce the influence of outliers that usually occur at border regions of the specimen and are off by a large magnitude when compared to the rest of the image, the data was normalized using the 0.5% and 99.5% percentile of the image intensities and standardized afterwards. Furthermore, to be able to train the network without special restrictions due to the specimen geometries and to facilitate the experiments’ procedure and support interchangeability of materials, the data was center cropped by a rectangle with a side length of 72 px. This size was determined based on the S245 geometry of the DX54D material, and the largest possible inner rectangle. Since the aim of the study is to identify the localized necking based on the material changes, the dark homogeneous border regions must be avoided, to prevent the network from learning features based on these curved border regions, rather than the intensities that are included within each patch. The changing image size and the amount of the curved region included within the evaluation area would bias the network, as visualized in Figure 2.

### 3.2. Network Architecture

**Figure 3:**
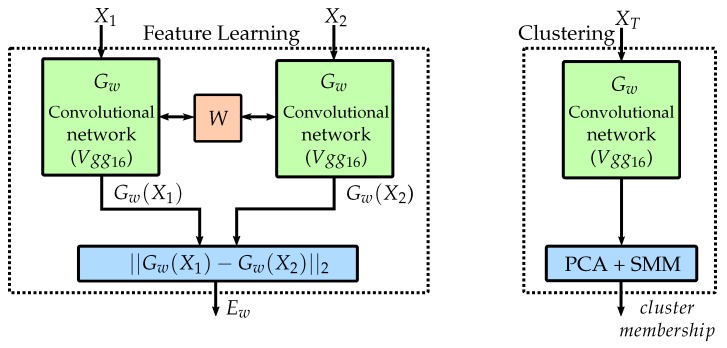
Supervised feature learning and unsupervised clustering, adopted from [25].

The Siamese network comprises two identical sub-networks, where each consists of the first 3 convolutional blocks of a VGG16 network [29] that was pretrained on a large-scale image database [30], rather than training the network from scratch with a limited amount of data. Additionally, to adapt to the new data, one dropout layer (0.5 dropout rate) between the two fully connected layers (512, 256 neurons) followed by one L2 normalization layer. The two CNN’s form a Siamese CNN architecture [25] as visualized in Figure 3 and depicted by feature learning, which is used for learning low-dimensional representations of the data, and to assess the images in a pairwise manner. Two inputs ($X_{1},X_{2}$) are evaluated simultaneously using an identical network structure ($G_{w}$) with shared weights (W). This setup allows a pairwise comparison between the two, L2 normalized, low-dimensional outputs of the network ($G_{w}\left(X_{1}\right),G_{w}\left(X_{2}\right)$), as different input images are assessed identically, based on a suitable distance measure.

### 3.3. Supervised Siamese Optimization

While other loss functions usually sum over individual samples, this loss function evaluates the input samples $X_{1},X_{2}$ in a pairwise manner, while learning the parameterized distance function $D_{W}$ using the Euclidean distance between the low-dimensional outputs of $G_{W}$.

(1) $$D_{W}\left(X_{1},X_{2}\right)={\left|\left|G_{W}\left(X_{1}\right)-G_{W}\left(X_{2}\right)\right|\right|}_{2}$$

The general loss function is described as,

(2) $$L\left(W\left(Y,X_{1},X_{2}\right)\right)=\left(1-Y\right)L_{S}\left(D_{W}\left(X_{1},X_{2}\right)\right)+YL_{D}\left(D_{W}\left(X_{1},X_{2}\right)\right)$$

where $\left(Y,X_{1},X_{2}\right)$ denotes a labeled input sample pair. The label *Y* refers to the extreme cases of the input sequence, where Y=0 if the samples are from the same class and Y=1 if the samples are from different forming phases. $L_{S}$ denotes the loss for samples of the similar phase while $L_{D}$ denotes the loss for dissimilar samples. These losses are designed such that minimizing *L* with respect to *W* leads to low values of $D_{W}$ if the samples are similar and high values otherwise.

The final loss function, the contrastive loss [31] is denoted as,

(3) $$L\left(W,Y,X_{1},X_{2}\right)=\left(1-Y\right)\frac{1}{2}{\left(D_{W}\left(X_{1},X_{2}\right)\right)}^{2}+\left(Y\right)\frac{1}{2}{max(0,m-D_{W}\left(X_{1},X_{2}\right))}^{2}$$

where *m* is a margin parameter that defines a radius around $G_{W}\left(X\right)$, the threshold distance for dissimilar pairs. Due to the incomplete nature of the data, as only the extreme cases are used for training, this parameter cannot be optimized and is naively set to 1.0.

### 3.4. Unsupervised Clustering

**Figure 4:**
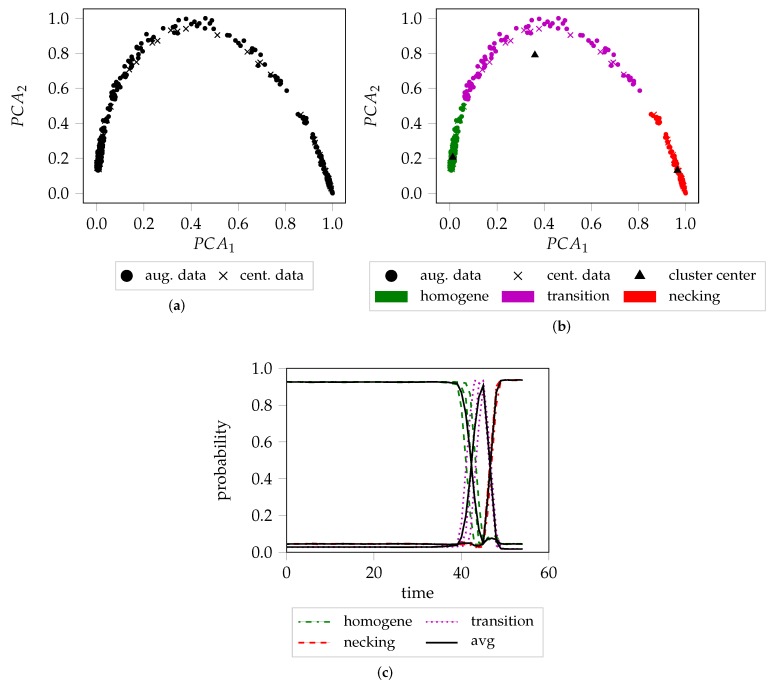
Exemplary procedure of the detection pipeline based on AA6014-S070: (**a**) Combined first two PCA components of the three forming sequences of AA6014-S070. (**b**) Same data as color coded cluster membership. (**c**) Cluster membership progression over time for each of the three sequences and their average.

So far the network is trained and optimized to create low-dimensional manifolds that support the optimal separation of the extreme cases of forming sequences. Even though, the network never saw a complete forming sequence, leads to the extraction of discriminative features from complete forming sequences. Hence, it may be used to assess and cluster the individual frames of video sequences using the low-dimensional feature representations. Principal component analysis (PCA) is applied to further reduce the dimensionality of the manifolds to two dimensions, facilitating the unsupervised clustering. The first two components were chosen, across all different geometries and materials, as they cover more than 95% of the variance of the data. In contrast to the training process of the network, where geometries and loading conditions of different materials were combined to increase the amount and variance of the data, to enable the network to learn generalized feature representations that describe necking, the individual geometries and materials are not combined during the assessment of the held-out data. This reduces the available information and number of data points that may be used to cluster the data. An increase in the density of the distributions is achieved by artificially augmenting the data, e.g., by cropping randomly, flipping, translating (by 5 px) and rotating (by 15 deg.) each image. The effect of such transformations is well described and visualized in [31]. For evaluation of each geometry of the held-out material, the three complete sequences of this geometry, depicted by $X_{T}$ are used by the network to create the manifolds, while after PCA dimensionality reduction, the components are clustered and described by distributions (cf. Figure 3 depicted by clustering), using Student’s t Mixture Models (SMM) [26]. These models are more robust to outliers compared to Gaussian Mixture models as shown in medical image segmentation [32] or registration [33]. This procedure enables the forming sequences to be described as probabilities, wherein, the probabilities represent the membership likelihood of the center cropped unaltered images at each time point, to the clusters of the mixture model. The clusters in turn represent distinct phases of the forming process, thereby enabling the frames of sequences to be classified into the same. The complete “unsupervised” pipeline is visualized in Figure 4. Figure 4a shows the unclustered PCA reduced features of the center cropped and augmented data of three trials of AA6014-S070. Figure 4b depicts the same data after being clustered with SMM, whereas Figure 4c visualizes the individual probability progression (with respect to mixture component membership) of each of the three sequences.

## 4. Experiments

Multiple experiments are conducted to analyze generalization of the method from one material to another, and to evaluate the possibility of validating metallographic examination results by categorizing individual images of video sequences without defined localization regions.

### 4.1. Leave One Material Out Cross Validation (lomocv)

**Figure 5:**
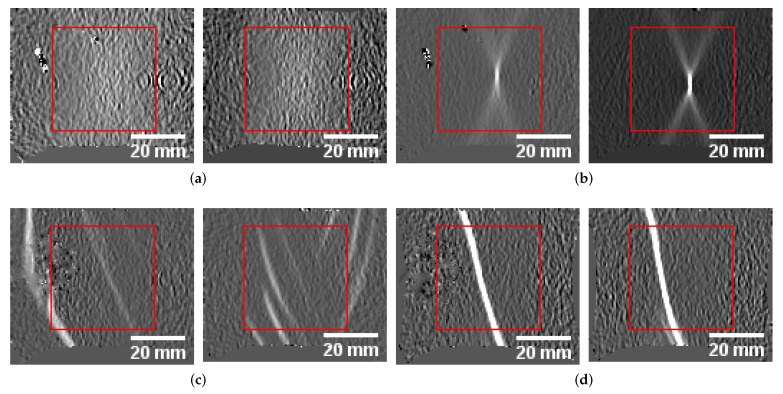
Comparison of homogeneous and localization phase cases of DP800-S050 and AA5182-S050: (**a**) DP800 homogeneous. (**b**) DP800 localization. (**c**) AA1582 homogeneous. (**d**) AA1582 localization.

AA5182 was not considered in this experiment, as its behavior and the appearance of its images are significantly different compared to the other materials and hence would deteriorate learning. During forming AA5182 develops shear bands at random locations that seem comparable to the necking behavior of the remaining materials, as visualized by the examples of the homogeneous and inhomogeneous forming phase of DP800-S050 and AA5182-S050, shown in Figure 5. The generalization and transferability of knowledge to unseen data is assessed with this experiment. One held-out material is evaluated after training the network with all geometries, of all materials, except the held-out one. Two thirds of the sequences are used as the training set, while the remaining sequences are used as the validation set. To generate an FLC for the held-out material, its sequences are processed by the network to generate low-dimensional representations. The determination of the actual strain values is carried out geometry wise. Three processed sequences per geometry are clustered using the described unsupervised framework, while the cross sections of the probability progression curves of the individual clusters (50% probability) are used as look up time points for the actual strain values.

### 4.2. Leave One Sequence Out Cross Validation (losocv)

The data used to train the network is restricted to one geometry and material. Two thirds of the data per geometry are used as training and validation set, while the remaining sequence serves as test set. This way it is possible to assess the loading conditions separately and at the same time assess the generalization to unseen, comparable data.

### 4.3. Overfit

All three video sequences per geometry are included in the training set and hence define a theoretical upper bound of the FLC. Of course, no generalization is assessed following this set up. In general, an overfit to the training data is invalid in pattern recognition approaches as it would not generalize to unseen data, but seems valid in the application of defining a forming limit based on three sequences for the generation of the FLC.

### 4.4. Metallography

This experiment assesses the location independence, as it evaluates multiple video sequences of forming processes of sheet metals that have not been deformed until fracture. The aim of this procedure was to stop the forming process close to the onset of localized necking, and hence to investigate the presence of material fatigue, based on metallographic examinations. The proposed method is used to validate these examination results and to ascertain if it is possible to assign frames to failure clusters without a well-defined localization region. Such an evaluation is not afforded by existing techniques, as they all require a priori definition of a localization area. As a result, of this procedure, strain path evaluations would be rendered unnecessary. Again, the evaluation is conducted using the held-out material.

Within all experiments, the 50% intersection of the homogeneous forming phase and transition phase as described in Figure 4, and subsequent the intersection between the transition phase and localization phase, serve as look up time points for the strain values to create the FLC. The network is implemented in Keras [34] a high-level API of the TensorFlow framework [35]. The network was trained with a learning rate of 0.0001 in case of the losocv experiment, whereas in case of lomocv and overfit experiment a learning rate of 0.00001 was used. All experiments employed the Adam optimizer [36] for contrastive loss minimization. The Adam optimizer was chosen due to its ability to estimate adaptive learning rates based on the observed data, which has been demonstrated to improve convergence when training deep neural networks.

## 5. Results and Discussion

### 5.1. Comparison of Experimental Results

**Figure 6:**
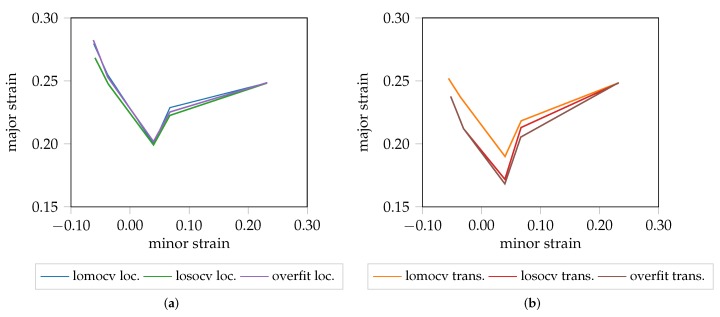
Different FLC candidates of DP800 (1.00 mm). (**a**) localization. (**b**) transition.

**Figure 7:**
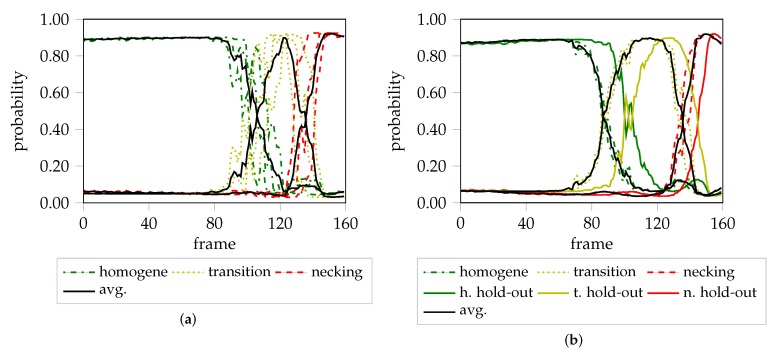
Differences between the lomocv and losocv experiment for DX54D-S060 (0.75 mm). (**a**) Probability progression for all three hold-sequences (losocv). (**b**) Probability progression for one hold-out sequence vs. the training sequences (losocv).

A graphical representation of the localization and transition FLC candidates of DP800 are visualized in Figure 6. The different evaluation approaches investigated are consistent when comparing the localized FLC candidate, independent of the strategy being used for training the network. This is reasonable, as the necking behavior is comparable throughout all materials except AA5182 and especially true when training the network using information from just one material and one geometry as in losocv and the overfit experiment. However, in the case of lomocv, the network never saw any sequence of this material, but is still able to determine the onset of localized necking, inferred from the learned necking behavior of the remaining materials (AA6014, DX54D). A slightly different picture arises when comparing the transition FLC candidates. The losocv and overfit experiment still show high correlation, whereas the candidate of the lomocv experiment is less conservative. One explanation for this rather large difference is the limited amount of data that is available in case of the losocv and overfit experiment. Additionally, if one of the three sequences is not comparable to the remaining ones, the proposed approach will not generalize to this sequence and hence the determined distributions may not be suitable for the held-out sequence. One example for this behavior is visualized in Figure 7 for DX54D-S060 (0.75mm), where Figure 7a depicts the result of the lomocv experiment. Here, the curves for the transition phase for all held-out sequences are closer to each other, while in Figure 7b the solution is biased towards the training sequences, as the held-out sequence seems displaced in comparison to the average of the training sequences.

This especially occurs, when artifacts as visualized in Figure 5 are not consistently present in all data sets, as these affect the standardization procedure, or if the localization behavior is very different from the training data set. However, this effect may be reduced by increasing the number of forming sequences per geometry that are used to train the network. In the case of the lomocv experiment, a lot more data is used to train the network, which leads extraction of more generalized features, and hence a less conservative transition candidate. However, the negative effect of artifacts may also deteriorate the result of the lomocv approach, when the sequences of the held-out material contain artifacts that are a lot different from the ones in the training set. Overall it seems beneficial to pursue a leave one material out approach rather than performing a leave one sequences out setup.

**Figure 8:**
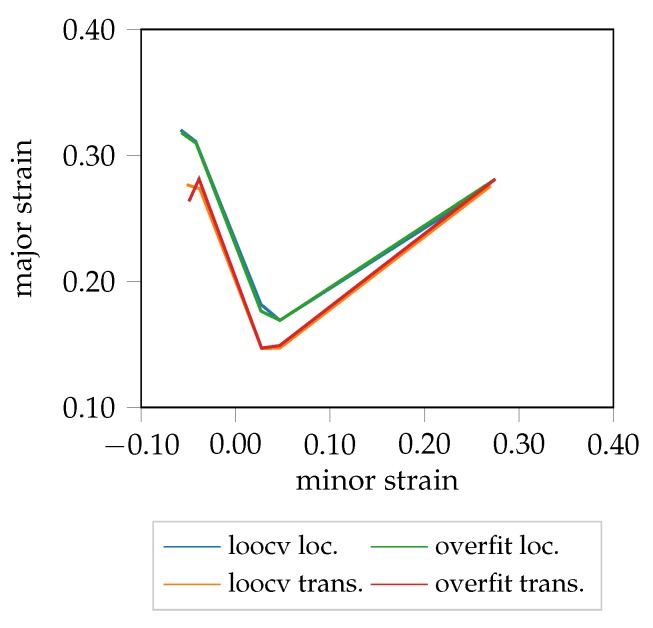
FLC candidates of AA5182.

Despite the availability of a limited amount of data, the proposed method generalizes to more complex materials such as AA5182, as visualized in Figure 8. Here, it additionally may be beneficial to increase the size of the squared evaluation area and change its shape to a rectangle (cf. Figure 5c), to cover the available image information close to the border of the specimen. In general, a geometry dependent evaluation size would be preferable, but would render the training process of the network more difficult, and would require more thought into designing the network architecture appropriately. Furthermore, it would decrease the amount of available data per evaluation area and hence would negatively affect training of the network.

### 5.2. Comparison with State of the Art

**Figure 9:**
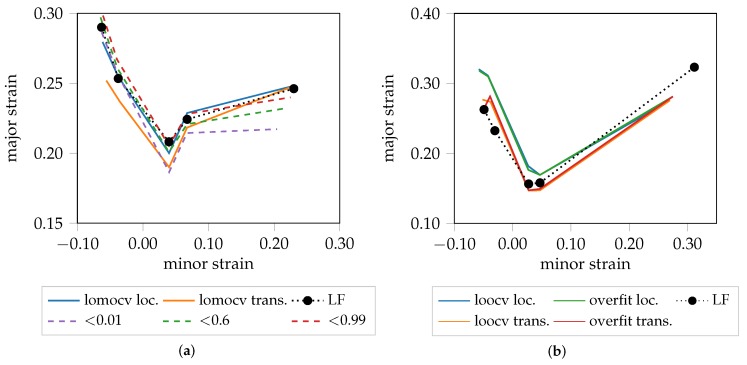
Comparison with state of the art for DP800 (**a**) and AA5182 (**b**).

The “line-fit” method (LF) as well as the unsupervised one-class SVM approach of Part 2 are compared to the method proposed in this study. The first approach focuses on a small heuristically defined evaluation area, and uses the reduction of material thickness to define the onset of necking. The second approach exploits more information, as it analyzes all principal strains simultaneously in multiple square evaluation areas in the vicinity of the maximum strain. Edge orientation information based on HoG features is extracted and classified using the one-class SVM coupled with a Gaussian mixture models (GMM) approach. As a result, the FLC can be expressed in terms of failure quantiles and hence leads to a probabilistic FLC, allowing a comparison of the proposed FLC candidates with different certainty levels. As this method expects a stable necking zone, the comparison is only made for DP800. The probabilistic FLC candidate of the unsupervised one-class SVM approach is depicted by the probability quantiles in Figure 9a. To facilitate the comparison, the lomocv loc. curve representatively substitutes the other two experiments as they share comparable results. It is in line with the 0.99% and 0.6% quantile, and additionally corresponds to the results of the “line-fit” method. The depicted lomocv FLC candidates are rather conservative, since the intersections between the homogeneous/transition and transition/necking class are used as lookup time points for the strain values and correspond to 50% probability. However, quantile profiles as presented in part 2 would also be possible, but were omitted for reasons of visualization. The lomocv trans. candidate is comparable to the 0.01% quantile candidate, while both are well below the results of the “line-fit” method. In general, the width of the transition distribution depends on the material and evaluated geometry, e.g., a broader transition phase is expected for DX54D-S060 (cf. Figure 7a), compared to the transition phase of AA6014-S070 (cf. Figure 4c).

**Figure 10:**
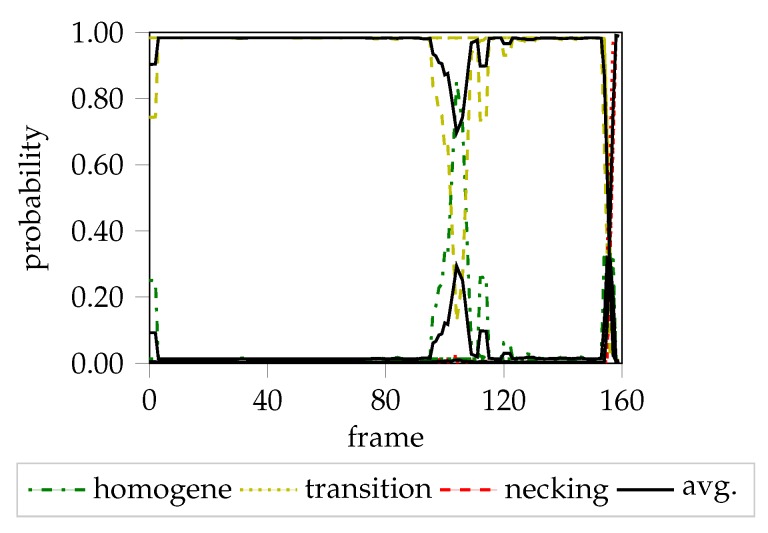
Probability progression of DP800-S245.

The homogeneous/transition intersection lookup for S245 was omitted in all experiments, since it tends to not occur within this geometry, or is very close to the necking lookup time point or even superimposed by the other two phases (cf. Figure 10). For the AA5182 material, the localized necking candidates are above the “line-fit” candidates in all but the S245 geometry (cf. Figure 9b), while the transition candidates are found slightly below the “line-fit” method, at least in near plane-strain conditions.

### 5.3. Metallography–Strain Path Quantification

**Figure 11:**
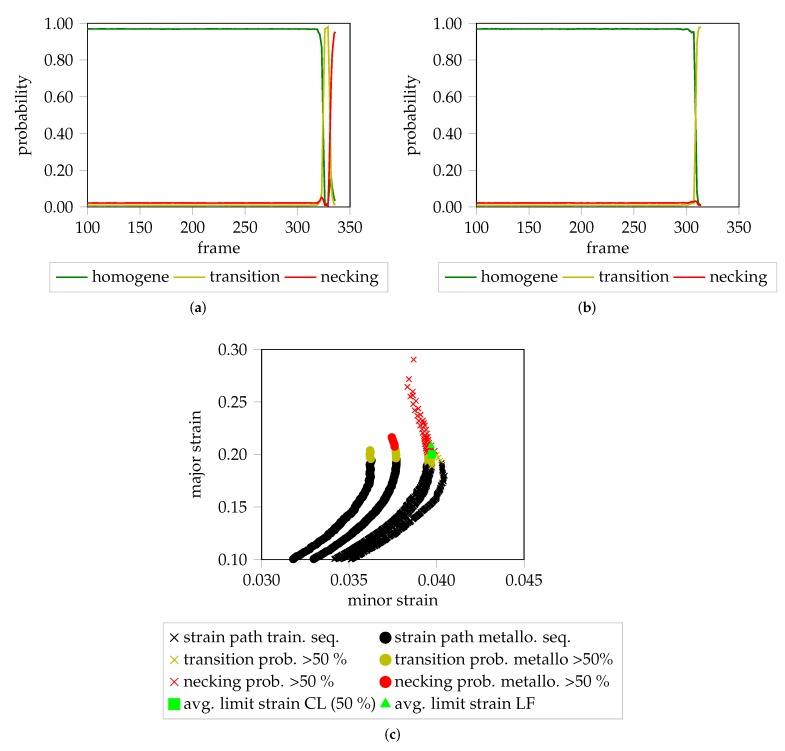
Color coded probability progressions and strain paths of two sequences of DP800-S110 that have not been deformed until fracture in comparison to the strain paths of the training sequences: (**a**) Probability progression of the first trial. All three classes are assigned to the individual frames of the forming sequence. (**b**) Forming of the trial was stopped within transition phase. (**c**) Color coded strain paths of prematurely stopped forming sequences and training sequences. The classified avg. limit strain (CL) is variable depending on the selected probability threshold.

Metallographic investigations are used to describe the micro-structure of materials qualitatively and quantitatively. Therefore, if forming processes are stopped early, it is possible to investigate the progress and status of formed specimen. Hence, if the forming process was recorded with a measurement system, macroscopic descriptions using the strain distributions of the last frame of the sequence correspond to the microscopic characteristics of the metallographic examination. With a multitude of experiments and small enough steps regarding punch progression it is possible to describe material behavior and ultimately the onset of localized necking in more detail (cf. [10] for DC04 and [11] for DP800). However, if the forming process was stopped before the localized necking occurred, a direct comparison with specimen that have been deformed until fracture may not be possible, as the absence of localization effects makes it impossible to find a well-defined evaluation area to create valid strain paths or image regions that can be used for comparison. The method proposed in Part 2 can evaluate image regions but the time-dependence in the cluster step limits the transferability due the requirement of sequences to have a fixed length. Using the proposed method, distributions are estimated based on the trials that have been deformed until fracture. This allows to infer the current cluster membership for a frame of an incomplete formed specimen to be inferred, based on the center cropped image information. This approach is location independent and enables comparisons in a manner independent of the presence/absence of localization effects, and could be used in on-line forming processes, to stop the forming procedure. Two sequences of prematurely stopped forming trials of DP800-S110 are visualized in Figure 11a,b with individual probabilistic cluster memberships per frame. The first sequence reaches the localized necking phase, whereas the second forming process was stopped within transition phase. The strain paths (cf. Figure 11c) are created based on the strain distribution using the top 90% of thinning as threshold and color coded using a 50% threshold for each class. Additionally, the determined onset of localized necking according to the proposed classification method (CL) and the “line-fit” method are highlighted.

## 6. Discussion

Nakajima-based forming processes induce patterns on the surface of sheet metal materials and within the strain distributions when using a measurement device. In part 1 a supervised classification algorithm with expert knowledge/annotations was introduced, leading to good agreement between experts and classification results. Part 2 introduced an unsupervised classification algorithm, without expert annotations, based on conventional pattern recognition that evaluates edge information within small rectangular patches near maximum strain values using a one-class SVM with HoG features. In general, it led to consistent results throughout the experiments conducted, and introduced a probabilistic FLC. The main idea of this procedure was to introduce well-established pattern recognition methods and specific features that evaluate edge responses to support the hypothesis that localized necking correlates with a sudden increase/decrease of principal strains within strain distributions. As long as the hypothesis is correct, the method seems suitable as a base line approach that enables the estimation of multiple degrees of necking certainty using the FLC quantiles.

However, it has four disadvantages: (1) location dependency, due to evaluation only in the vicinity of the maximum strain area; (2) time-dependency, as it requires specimen to be formed until fracture and hence renders a comparison with prematurely stopped forming processes impossible; (3) predefined features limited to edge information; (4) transfer of knowledge to other materials or forming processes is not possible (i.e., it does not permit generalization to new materials/processes).

The proposed method overcomes these limitations in a two-step approach. First a Siamese CNN is trained in a supervised manner, where only the extreme cases of the homogeneous and inhomogeneous forming phase (begin/end of forming process) are used to train the network. These images are separated optimally by minimizing the contrastive loss function, while augmentation is used to increase the data amount throughout several experiments. In the second step, complete forming sequences, not only the extreme cases, are assessed by the network and transformed into low-dimensional representative manifolds. Using PCA and SMM, these manifolds are clustered in an unsupervised manner into three distributions corresponding to the three phases, homogeneous, transition and localized necking. This procedure overcomes the mentioned limitations: (1) location dependency is reduced as a consequence of using the maximum possible square evaluation area of all materials; (2) the overall framework is now independent of time, by replacing the one-class SVM with SMM, allowing assessment of individual frames of incomplete forming sequences coupled with strain paths; (3) optimal features are learned by the Siamese CNN, and are not limited to edge information; (4) transfer of knowledge from one material to another is possible as shown with the lomocv experiment, enabling on-line supervision of unknown materials during forming processes; and (5) generalization of the method to materials with complex forming behavior such as AA5182, with limited data is possible as shown within the overfit and losocv experiment. However, accommodating measurement artifacts and defect pixels remains challenging, as their presence or absence may bias training of the network or negatively affect clustering of the data.

## 7. Conclusions

In this study, a semi-supervised classification approach for the detection of the onset of localized necking was proposed. It comprises two steps: (1) a supervised, data-driven, feature learning part using a Siamese CNN, where only the extreme cases of the forming sequences were employed; and (2) an unsupervised clustering part, used to group the remaining frames of the sequence as belonging to the homogeneous, transition, or inhomogeneous forming phase. The results achieved using the proposed approach are consistent with previous approaches. However, while the latter required design and selection of suitable handcrafted features and a priori definition of the evaluation area, the main advantages of the proposed approach are its location and time independency. Consequently, the proposed method enables on-line identification of a distinct time point for the onset of localized necking, and could be used to stop forming processes, using the acquired image information. The question of what exactly is localized necking in terms of the strain distribution remains unanswered. So far, the top 90% of thinning or other heuristics are used to define the region regarded as localized necking. Future work could investigate a data-driven approach to learn segmentations of regions representative of localized necking. 

## Figures and Tables

**Table 1:** Material properties of the investigated materials [13] including AA5182.

Material	t_0_ (mm)	n	YS (MPa)	TS (MPa)	UE (%)	r_0_	r_90_	Frequency (Hz)	Punch Velo-City (mm/s)
DX54D	0.75/2.00	0.23	164–170	297–322	22–23	1.80	2.22	40/20	1.0/1.5
DP800	1.00	0.16	465	775–797	14–16	0.76	0.90	40	1.0
AA6014	1.00	0.24	140–143	244–239	21–23	0.69	0.67	15	1.0
AA5182	1.00	0.32	130–132	265–275	20–21	0.72	0.67	40	1.0

**Table 2:** Database per material with process parameters.

Material (Thickness mm)	# Images/# Homog./# Neck.	Frequency (Hz)	Punch Velo-City (mm/s)	Available Geometries
DX54D (2.00)	80/20/5	20	1.5	S030, S060, S080, S100, S125, S245
AA6014 (1.00)	55/15/3	15	1.0	S050, S060, S080, S100, S110, S125, S245
DP800 (1.00)	160/20/3	40	1.0	S050, S060, S110, S125, S245
DX54D (0.75)	160/20/5	40	1.0	S050, S060, S070, S080, S090, S100, S110, S125, S125, S245
AA5182 (1.00)	160/30/3	40	1.0	S050, S060, S110, S125, S245
